# The evolution of plasmid-carried antibiotic resistance

**DOI:** 10.1186/1471-2148-11-130

**Published:** 2011-05-19

**Authors:** Fabian Svara, Daniel J Rankin

**Affiliations:** 1 Institute of Evolutionary Biology and Environmental Studies, University of Zürich, Building Y27, Winterthurerstrasse 190, CH-8057 Zürich, Switzerland; 2 Swiss Institute of Bioinformatics, Quartier Sorge, Bâtiment Génopode, CH-1015 Lausanne, Switzerland

**Keywords:** plasmid, resistance, antibiotic, horizontal gene transfer, mobile elements

## Abstract

**Background:**

Antibiotic resistance represents a significant public health problem. When resistance genes are mobile, being carried on plasmids or phages, their spread can be greatly accelerated. Plasmids in particular have been implicated in the spread of antibiotic resistance genes. However, the selective pressures which favour plasmid-carried resistance genes have not been fully established. Here we address this issue with mathematical models of plasmid dynamics in response to different antibiotic treatment regimes.

**Results:**

We show that transmission of plasmids is a key factor influencing plasmid-borne antibiotic resistance, but the dosage and interval between treatments is also important. Our results also hold when plasmids carrying the resistance gene are in competition with other plasmids that do not carry the resistance gene. By altering the interval between antibiotic treatments, and the dosage of antibiotic, we show that different treatment regimes can select for either plasmid-carried, or chromosome-carried, resistance.

**Conclusions:**

Our research addresses the effect of environmental variation on the evolution of plasmid-carried antibiotic resistance.

## Background

The emergence of antibiotic resistance in pathogenic bacteria, both in hospital and community-acquired infections, represents a significant public health problem [1-5]. Bacterial cells are capable of transferring genes horizontally. This DNA transfer can take place in three ways, through plasmids, phages, or uptake of naked DNA [6]. Plasmids are extra-chromosomal pieces of DNA, which are capable of replicating independently of the genome, and are particularly important in the spread of antibiotic resistance genes. Plasmids have been directly implicated in the acquisition of resistance to many antibiotics [7-14]. This is particularly problematic since plasmids can cross many species and genus barriers, and the rate of plasmid transfer has even been shown to increase in more heterogeneous communities [15]. Plasmids thus allow resistance to spread and persist in niches that are not necessarily subject to antibiotics [16].

**Figure 1:**
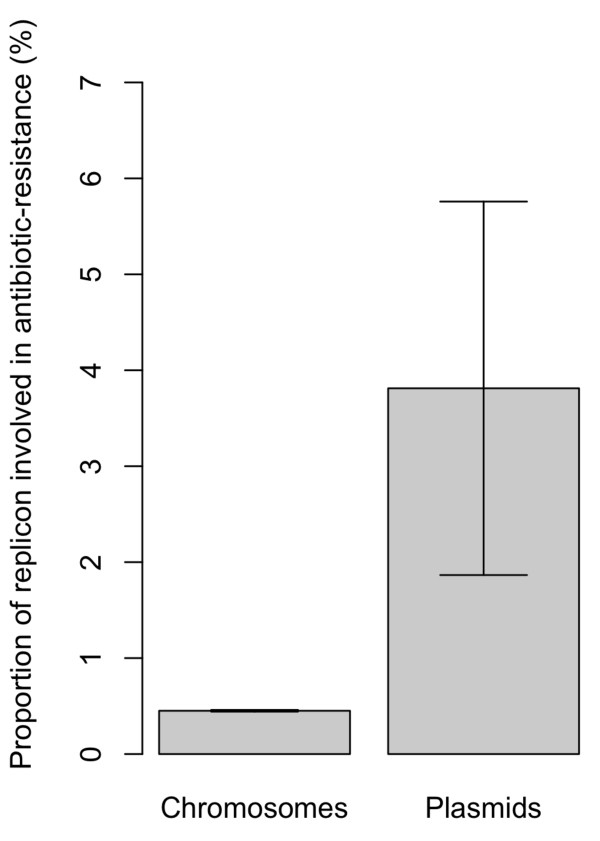
**Percentage of the genome among 37 *Escherichia *and *Shigella *strains which code for genes related to antibiotic resistance**. This shows that antibiotic resistance genes are over-represented on plasmids. Bars show the mean percentage (±s.e.) in all replicons (either on the chromosome, *n *= 37, or on plasmids, *n *= 53, including 38 plasmid genomes which contained no antibiotic resistance genes). The genomes are from 37 *Escherichia *and *Shigella *strains, including all chromosomal and plasmid genes in each genome (data obtained from the NCBI at http://www.ncbi.nlm.nih.gov/genomes/). There were a total of 174,862 genes, with 170,709 on all 37 chromosomes and 4,153 on all 53 plasmids. For comparison with known genes involved in antibiotic resistance, the "Antibiotic Resistance Database" [http://ardb.cbcb.umd.edu/- [62]] was used to identify genes involved in antibiotic resistance (homologues were identified using an E-value of 10^-10 ^and percentage of sequence identity between 60-95%). From this, there were a total of 817 genes involved in antibiotic resistance, with 765 carried by the bacterial chromosomes (and 0.48% of all chromosomal genes) and 52 carried by plasmids (1.25% of all plasmid genes). A *χ*^2 ^test revealed that antibiotic resistance genes were significantly over-represented on plasmids, compared to on the bacterial chromosome (Pearson's *χ*^2 ^with Yates' continuity correction: *χ*^2 ^= 54.6, *p *< 10^-12^, df = 1).

In *Escherichia *and *Shigella *strains, a larger proportion of the genome of plasmids codes for antibiotic resistance than that of the chromosome (Figure 1 - see figure legend for details). However, like all mobile genetic elements, plasmids face the fundamental trade-off between the cost of their mobility and the advantage imparted by their accelerated spread [e.g. [17]]. Plasmids can be seen as genomic parasites, as they can persist despite the costs they impose on their bacterial hosts [18-20]. Previous models have examined the conditions under which plasmids can be maintained in the population [18,19,21,22]. It is generally accepted that parasites can persist if their *R*_0_, their per-capita rate of increase in a susceptible population of hosts, is greater than 1 [23-25]. In the case of parasitic plasmids, which do not carry beneficial traits, and inflict a net cost on their host, this means that the rate of horizontal transfer must exceed the cost that they impose on their host [19]. Despite being fundamentally parasitic by nature, plasmids often carry traits which are beneficial to a host, or a host's neighbours [18,20,26-31]. These dynamics will likely be modulated by environmental conditions, such as antibiotic treatment. Both the dosage of antibiotics and the interval between treatments is likely to affect the evolution of plasmid-borne resistance [e.g. [18,21]]. Here we examine the role that these parameters will have on the evolution of plasmid-borne resistance. We address this question with a simple model of antibiotic resistance in a population exposed to different treatment regimes.

## Methods and Results

### Basic model

We start off with a population of wild-type cells ("free" cells) that do not carry plasmids or antibiotic resistance genes. Assuming logistic growth, with a growth rate of *r*, environmental antibiotic concentration *A*, the dynamics of the wild type *n*_*F *_cells are:(1)

The density-dependent death rate is given by *a*. This *per capita *death rate a depends on the density of cells in the population. In a population of wild-type cells this is simply *n*_*F*_, since there are no other cell types. We assume an antibiotic-induced fitness cost *Am*. Antibiotics can have two actions on a cell: they can either kill the bacteria (bactericidal antibiotics such as penicillin) or they can prevent reproduction (bacteriostatic antibiotics, such as tetracycline). Our model is general and does not differentiate between the two and could thus apply to either. From equation (1), at equilibrium, d*n*_*F*_/d*t *= 0, there are two equilibrium points, *n*_F_* = 0 and *n*_F _* = (*r-mA*)/*a*. This implies that the antibiotic will drive the host population extinct if *mA *>*r*. Thus, the higher the concentration of antibiotic, the more likely it is that wild-type will be eradicated.

To keep our model simple, we simply focus on cells which are either resistant to the antibiotic, or not, and where the gene is localized in the genome (on a plasmid or a chromosome). We therefore assume that genes for resistance against an antibiotic can be carried either by the chromosome or by a plasmid. As such, our model does not make any other assumptions about the genetics of the systems, such as compensatory mutations or the number of mutations needed to confer resistance to the antibiotic. We assume that plasmids can transfer horizontally by conjugation at a rate *β*. The dynamics of wild-type cells now becomes:(2)

Details of parameters used in all models are given in Table 1. The total population density is given by *N *= *n*_F_+*n*_P_+*n*_C_+*n*_CP_, where *n*_*P *_is the density of wild-type cells which are infected by a plasmid carrying a gene for antibiotic resistance and *n*_*CP *_is the density of cells which both carry a gene for antibiotic resistance on the chromosome and are infected by a plasmid carrying a gene for antibiotic resistance. We assume that cells which carry a gene for antibiotic resistance are fully immune to the effects of the antibiotic (although we later relax this assumption).

**Table 1:** Parameters used in the model

Parameter	Description
*n*_*F*_	Density of wild-type/plasmid-free cells ('F' in figures)
*n*_*C*_	Density of cells with resistance on the chromosome ('C' in figures)
*n*_*P*_	Density of cells infected with a resistance-carrying plasmid ('Plasmids' in figures)
*n*_*B*_	Density of cells infected with a non-coding plasmid ('B' in figures)
*n*_*CP*_	Density of cells with resistance both on the chromosome and on a plasmid ('CP' in figures)
*n*_*CB*_	Density of cells infected with a non-coding plasmid, and with resistance on the chromosome ('CB' in figures)
*N*	Total density of cells *N *= *n*_*F*_+*n*_*C*_+*n*_*P*_+*n*_*B*_+*n*_*CP*_+*n*_*CB*_
*A*	Concentration of antibiotic
*l*	Rate of decay of antibiotic
*θ*	Treatment dosage of antibiotic
*τ*	Interval between antibiotic treatments
*r*	Intrinsic per-capita growth rate of cells
*a*	Extrinsic density-dependent death rate of cells
*m*	Death rate of non-resistant cells, due to antibiotic
*c*_*C*_	Cost of antibiotic resistance when gene carried on chromosome
*c*_*P*_	Cost of antibiotic resistance when gene carried on plasmid
*x*	Cost of plasmid carriage
*β*	Rate of horizontal transfer of plasmids
*s*	Rate of segregation

Antibiotic resistance is likely to inflict a cost on the carrying individual [32-37], which we denote *c*_*C *_in the case of chromosome-carried resistance and *c*_*P *_in the case of plasmid-carried resistance. The dynamics of chromosome-carried resistance genes are:(3)

Building on previous models of the dynamics of plasmid carriage [18,19,38,39], and assuming that plasmids are fundamentally genomic parasites [18-22], we assume that there is a cost *x *to a cell from plasmid carriage. The dynamics of plasmid-infected cells are therefore:(4)

Cells with resistance on the chromosome can also be infected by plasmids. In this case there will be multiple copies of the gene and the cell will pay the cost of carrying both genes (i.e. *c*_*C *_+ *c*_*P*_). The dynamics for such cells are:(5)

### Analytical results

From equations (1)-(5), we can look at the conditions under which resistance (either carried by plasmids or coded on the chromosome) will invade. The condition under which a cell with resistance on the chromosome will invade, if wild-type cells are at equilibrium *n*_F_* = (*r-mA*)/*a *(and if there are no other cells in the population), is if

This occurs if the net benefit (*Am*) from being resistant to the antibiotic is greater than the costs *c*_*C *_of resistance. When the wild-type cells are at equilibrium (and there are no other cells in the population), a plasmid carrying the resistance gene will invade if

This shows that lower costs (in terms of *x*, *s *and *c*_*P*_), higher rates of transfer of the plasmid given by *β*(*r*-*mA*)/*a*, or greater impact of the antibiotic on wild-type cells (given by *mA*) will all favour the invasion of plasmid resistance.

Plasmids will be selected to carry antibiotic resistance genes if the fitness of cells with resistance on a plasmid is greater than the fitness of cells with resistance on a chromosome. Plasmid-bearing cells will grow faster than cells with resistance on the chromosome when both are rare (i.e. *n*_*P*_→0, *n*_*C*_→0, *n*_*CP*_→0) if the following condition is fulfilled:(7)

From inequality 7, we find that the plasmid will be favoured if(8)

This shows that plasmids cannot invade the population if there are no wild-type cells (i.e. *n*_*F *_= 0). As the antibiotic will kill wild-type cells, cells with resistance on the chromosome will be favoured over cells with resistance on a plasmid if the antibiotic has substantially reduced the density *n*_*F *_of wild-type cells. Plasmid-carried resistance will also be favoured over chromosome-carried resistance if the costs of expressing a resistance gene on the chromosome *c*_*C*_, is greater than the cost of expressing a resistance gene on a plasmid *c*_*P*_.

If the antibiotic fails to completely eradicate the population of wild-type cells, a wild-type population with no plasmids will have an equilibrium density of *n*_F_* = (*r*-*Am*)/*a*, assuming that the concentration of antibiotics is not enough to drive them extinct (which requires that *r *>*Am*). From this, plasmid-borne resistance will thus be favoured over chromosomal resistance when

This implies that higher per-capita growth rate, or low doses of antibiotic, will favour plasmid-borne resistance. Consequently, plasmids will be favoured when the wild-type density is not much constrained by antibiotic-induced mortality. Plasmids will have a faster growth rate than wild-type cells (cells without any resistance gene or plasmid) if

which will occur when

When the wild-type cells are at equilibrium *n*_F_* = (*r*-*Am*)/*a *then this criteria becomes:

This shows that higher transfer rates *β *of the plasmid, as well as a greater effect of the antibiotic, or lower overall costs from carrying the plasmid (*x *+*s *+ *c*_*p*_) will all result plasmid-carried resistance have a greater over all rate of spread than wild-type cells. Interestingly, greater growth rates *r *will also favour plasmid-carried resistance to have a larger growth rate than wild-type cells.

### Numerical analysis model

We now explore how different treatments select for antibiotic resistance to be carried either on a plasmid, or a chromosome, using numerical simulations of our system. We built a numerical simulation to investigate how dosage strength and interval affect whether resistance will be found on the host chromosome or a plasmid. We start with a population of wild-type cells at the carrying capacity (i.e. *n*_F _= 1). Following [40], we assume exponential degradation at a rate *l *for the antibiotic:(6)

**Figure 2:**
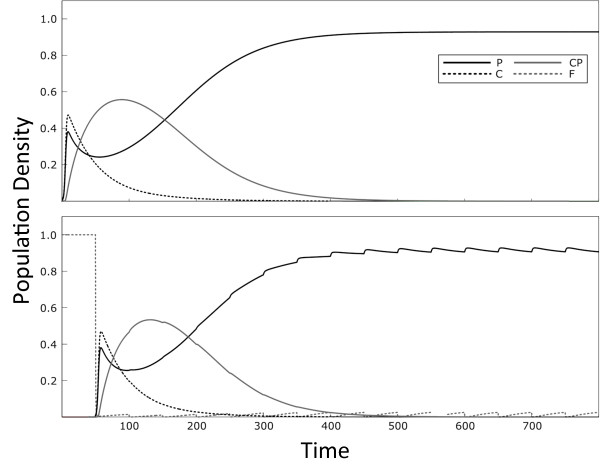
**Dynamics of the basic model (consisting of wild-type cells, cells with resistance on the chromosome and plasmid-carried resistance) in response to different treatment regimes**. "F" denotes wild-type cells, "P" denotes cells infected with a plasmid carrying resistance, "C" denotes cells with resistance on the chromosome and "CP" stands for cells with resistance on the chromosome that also carry a resistance plasmid. Parameters used are *r *= 1, *a *= 1, *β *= 0.006, *s *= 0.001, c_c _= 0.002, c_p _= 0.002, *x *= 0.002, *m *= 0.1 and *l *= 0.005. The dosage is 50 in both panels, while the treatment interval is *τ *= 5 time units in the top panel and *τ *= 50 time units in the bottom panel. Time in our model is scaled, and is thus measured in arbitrary units (AU).

We use the Euler method of integration with a time step of 0.001. Once the cells are at the equilibrium density, we then assume that the antibiotic is introduced, at a dosage of *θ*. We then assume that an amount *θ *of the antibiotic is re-introduced in regular treatment intervals of *τ *time-steps. At the beginning of the simulation, we assume that only 0.1% of cells contain the resistance gene, and these are carried either on a plasmid or on the host chromosome (i.e. *n*_C _= 0.001 and *n*_P _= 0.001 at the start of the simulation). We then run the simulation for 5,000 time-steps, or until the cells have reached a stable dynamical equilibrium. Examples of the dynamics of the model are given in Figure 2, illustrating the case where the interval is either 5 (top panel) or 50 (bottom panel).

**Figure 3:**
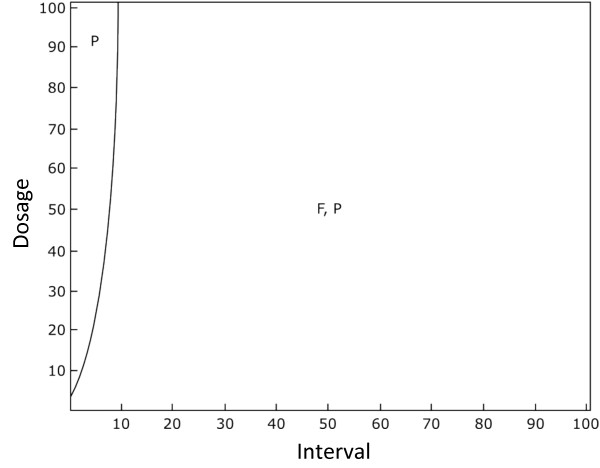
**The effect of antibiotic dosage intensity and the interval between treatments on the cell types persisting at equilibrium for the basic model (consisting of wild-type cells, cells with resistance on the chromosome and plasmid-carried resistance)**. Here, chromosomal resistance can be outcompeted by plasmid-carried resistance. "F" denotes wild-type cells and "P" denotes cells infected with a plasmid carrying resistance. Cell types that are present in the population at a density greater than exceeding 0.001 are shown. The plots were calculated by running the simulation for a number of parameter values for 5,000 time-steps. Lines were then smoothed by interpolation. Parameters used are *r *= 1, *a *= 1, *β *= 0.1, *c*_*c *_= 0.02, *c*_*p *_= 0.02, *x *= 0.05, *m *= 0.1, *s *= 0.001 and *l *= 0.5.

Figure 3 shows the qualitative outcome for a large set of treatment intervals and dosages. The boundaries were interpolated for increased clarity. Under the conditions shown in Figure 3, chromosomal resistance does not evolve, and that lower intervals between treatments, or high dosages of antibiotics, increasingly favour a population only consisting of plasmid resistance, as the wild-type cells are killed by the antibiotic. However, as shown with our analytical model, the strain that wins depends strongly on the transmission rate (Additional File 1). In the case that the transmission rate beta is low, chromosomal resistance will be favoured if the interval is short and the treatment dosage is high, while as the transmission rate increases, plasmids increasingly dominate across the population. These confirm the results of our analytical model and suggest that transmission is the most important factor promoting plasmid-carried resistance.

### Model with non-coding plasmids

Up to now, we used the simplifying assumption that plasmids would always carry resistance genes. It has been shown previously that plasmids which do not carry the focal gene can have a substantial impact on the invasion of horizontally-transferred genes [39]. While a significant proportion of the genome of plasmids is involved in antibiotic resistance, many plasmids contain no genes involved in antibiotics at all (Figure 1 - see figure legend for details). Thus, we now additionally consider plasmids which are infectious, but do not code for resistance, which we refer to as "non-resistant plasmids". These cells pay a cost of plasmid carriage, but do not carry resistance to the antibiotic, and are therefore susceptible to the antibiotic. The dynamics of wild-type, cells with resistance on the chromosome and cells with resistance on a plasmid now become:(9)(10)(11)

We assume that non-resistant plasmids are incompatible with resistance plasmids, and hence a cell can be infected by only one type of plasmid. The density of cells infected with non-resistant plasmids *n*_*B *_is given by:(12)

The density of cells which carry resistance on both the chromosome, and on a plasmid, is given by *n*_*CP*_. The dynamics of these cells are:(13)

Finally, we also assume that cells which have resistance on the chromosome can be infected with a non-resistant plasmid, and in this case the cell pays the cost *x *of bearing a plasmid and the cost *c*_*c *_of antibiotic resistance. The density of such cells is given by *n*_*CB *_and their dynamics is given by:(14)

In the basic model, plasmids were favoured due to their ability to transfer infectiously. In the absence of antibiotics, non-resistant plasmids will outcompete resistance plasmids, because resistance plasmids additionally pay the cost of antibiotic resistance gene expression *c*_*P*_. As in the basic model, we assume that the antibiotic degrades at an exponential rate described by equation 6. We analyse the system described by equations 9-14 using the numerical method described in the previous section, but here we start at the point where both *n*_*F *_and *n*_*B *_have reached equilibria (i.e. the population already consists of wild-type cells and non-resistant plasmids coexisting), before introducing a small number of cells with resistance on the plasmid, or on the chromosome, respectively (i.e. *n*_P _= 0.01, *n*_C _= 0.01, *n*_CP _= 0, *n*_CB _= 0 at the start of the simulation).

**Figure 4:**
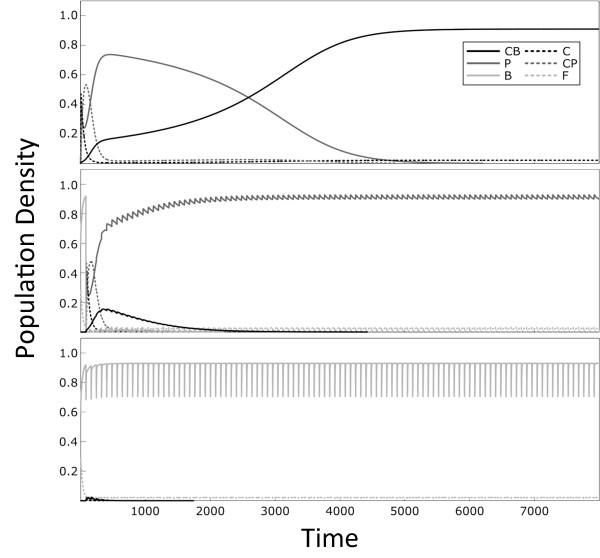
**Dynamics of the full model (consisting of wild-type cells, cells with resistance on the chromosome, plasmids carrying resistance and plasmids with no resistance) in response to different treatment regimes**. "F" denotes wild-type cells, "P" denotes cells infected with a plasmid carrying resistance, "C" denotes cells with resistance on the chromosome, "B" denotes cells infected plasmids which do not carry the resistance gene, and "CP" and "CB" stand for the chromosomally resistant cell types also carrying the respective plasmid. Parameters used are *r *= 1, *a *= 1, *β *= 0.1, *s *= 0.001, c_c _= 0.02, c_p _= 0.02, *x *= 0.05, *m *= 0.1 and *l *= 0.5. The dosage is 50 in the top two panels and 5 in the bottom panel, while the treatment interval is *τ *= 1 in the top panel and *τ *= 80 in the bottom two panels.

**Figure 5:**
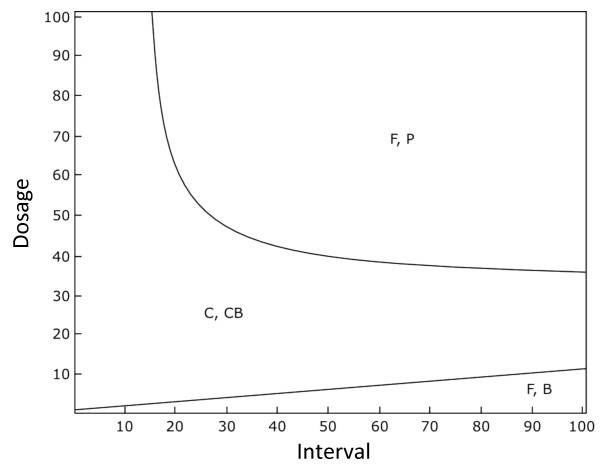
**The effect of antibiotic dosage intensity and the interval between treatments on the cell types persisting at equilibrium for the full model (consisting of wild-type cells, cells with resistance on the chromosome, plasmids carrying resistance and plasmids with no resistance)**. "F" denotes wild-type cells, "P" denotes cells infected with a plasmid carrying resistance and "C" denotes cells with resistance on the chromosome and "B" denotes cells carrying plasmids that do not code for resistance genes. Here, plasmid-carried resistance is favoured when dosage and interval between treatments are high. Cell types that are present in the population at a density greater than exceeding 0.001 are shown. The plots were calculated by running the simulation for a number of parameter values for 5,000 time-steps. Lines were then smoothed by interpolation. Parameters used are *r *= 1, *a *= 1, *β *= 0.1, c_c _= 0.02, c_p _= 0.02, *x *= 0.05, *m *= 0.1, *s *= 0.001 and *l *= 0.5.

Figure 4 shows examples of the dynamics that result for different treatment regimes. Figure 5 shows the conditions under which resistant plasmids persist under a wide range of different treatment regimes. This shows that plasmid-carried resistance is favoured under more intense, but less frequent, treatment regimes. Figure 5 shows that plasmids that carry resistance are outcompeted by cells with resistance on the chromosome at lower dosages and shorter treatment intervals (Figure 5). In contrast, non-resistant plasmids are favoured only when there is a low dosage of antibiotic applied with long intervals between treatments. Under weak treatments, the non-resistant plasmids outcompete plasmids carrying the resistance gene due to the costs *c*_*p *_of expressing the resistance gene. In general, the results of our extended model support our basic model, and confirm our predictions that plasmid-coded resistance will outcompete chromosomally-coded resistance under long-interval treatment regimes.

**Figure 6:**
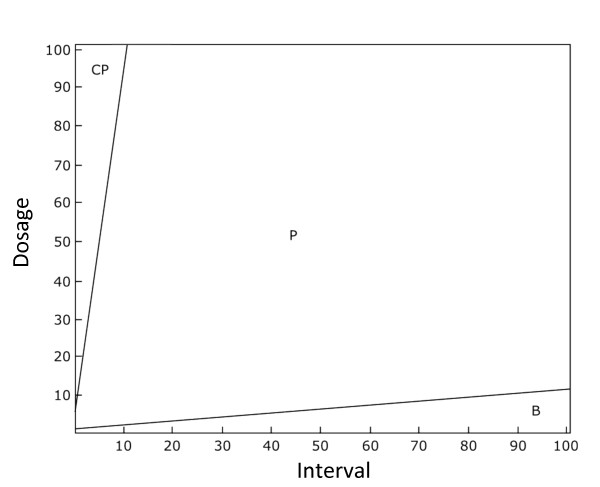
**The effect of antibiotic dosage intensity and the interval between treatments on the cell types persisting at equilibrium for the full model (consisting of wild-type cells, cells with resistance on the chromosome, plasmids carrying resistance and plasmids with no resistance) without segregation (i.e. *s *= 0)**. Here, plasmid-carried resistance is favoured over non-resistant plasmids at higher dosages, but shorter treatment intervals, but are favoured over chromosomal resistance at lower dosages but longer treatment intervals. This figure shows the case where all cells are susceptible to the antibiotic but where "C" cells suffer a mortality of *m *= 0.002, "P" cells suffer a mortality of *m *= 0.001 and "CP" cells have a combined mortality of 0.000002 (the product of the two mortalities). Wilt-type cells suffer a mortality *m *= 0.1. "F" denotes wild-type cells, "P" denotes cells infected with a plasmid carrying resistance and "C" denotes cells with resistance on the chromosome and "B" denotes cells carrying plasmids that do not code for resistance genes. Cell types that are present in the population at a density greater than exceeding 0.001 are shown. The plots were calculated by running the simulation for a number of parameter values for 5,000 time-steps. Lines were then smoothed by interpolation. Parameters used are *r *= 1, *a *= 1, *β *= 0.1, *c*_*c *_= 0.02, *c*_*p *_= 0.02, *x *= 0.05 and *l *= 0.5.

It is likely that cells that have resistance genes on a plasmid will be less susceptible to the antibiotic than cells which have resistance genes on a plasmid. To explore this, we looked at the case where all cell types could have differing levels of susceptibility. In this case, cells with resistance on the chromosome suffered a mortality of *m *= 0.002 per cell per unit of antibiotic, while cells with resistance on a plasmids suffered a mortality of *m *= 0.001 per cell per unit of antibiotic. If a cell carried the resistance gene both on the chromosome, or on a plasmid, we assumed the effective of having both genes would be additive, and the antibiotic-induced mortality of 0.000002 (i.e. the product of the two mortalities). As in the previous models, any cell which did not have either resistance gene suffered a per unit antibiotic mortality of *m *= 0.1. The results are plotted in Figure 6 (this is in comparison to Figure 5 as the comparison case where resistance is 100% effective). In this case, more intensive treatment regimes (i.e. high dosages, with short intervals between doses) favour a cells with both resistance on a plasmid and on the chromosome (Figure 6). This is in contrast to the case where resistance is 100% effective, where only cells carrying resistance on the plasmid persist at intensive treatment regimes.

We further explored our results for different parameter value combinations, such as the transmission rate (Additional Files 1 and 2), the segregation rate (Additional File 3), the respective cost of having resistance on a plasmid or a chromosome (Additional File 4) and the mortality cost *m *and the degradation rate *l *of the antibiotic (Additional File 5). All graphs can be found in the supplementary material. Our results generally held when changing these parameter values.

## Discussion

Plasmids are favoured when their rate of horizontal transfer outweighs the costs involved in plasmid carriage to a cell [19]. However, in the presence of less costly plasmids, the benefit of transfer disappears [39]. In our basic model in the absence of non-resistant plasmids, plasmids can persist as long as the rate of transfer is sufficient (Figure 3, Additional File 1). Our full model, incorporating non-resistant plasmids, shows that resistant plasmids outcompete non-resistant plasmids under higher dosages, and outcompete cells which resistance on the chromosome under longer intervals between treatments (Figures 5 and 6). This is due to horizontal gene transfer: wild-type cells are common when treatment is weak and their presence allows plasmids to invade. Resistance genes encoded by the host genome are favoured by treatment regimes that result in a continual presence of antibioitic. We can understand this intuitively by considering that the infectious maintenance of plasmids in a population consisting only of plasmid-carrying and wild-type cells requires the availability of plasmid-free cells. This is because carrying a plasmid and expressing resistance has a cost which has to be offset by the benefit of infecting susceptible cells. When susceptible cells are killed by antibiotics, plasmids suffer indirectly as they have fewer cells to infect. It is worth noting that our model assumes relatively efficient horizontal transfer, and thus our results may change if plasmids do not transmit themselves well. Our model would therefore predict that plasmids with lower transmission rates would not carry genes for antibiotic resistance. However the generality of our model of horizontal transfer of plasmids means that our findings will apply to other vehicles of gene transfer, such as phages.

We assumed that chromosomal- and plasmid-borne resistant types were phenotypically identical. This may not be the case [41] as changes in gene dosage can influence the resistance level [42-47]. We predict that higher gene dosage from multi-copy plasmids would enlarge the treatment space beneficial to plasmids, due to the additional resistance compared to chromosomally carried resistance genes. In the absence of antibiotics, hosts carrying multiple copies of resistance genes would be at a disadvantage due to the extra cost of producing more antibiotic resistance proteins. An important result of our study is to illustrate how the ecology of the bacteria, in this case the density of wild-type cells (mediated by the concentration of antibiotic), affects the conditions under which plasmid-borne resistance is favoured.

Our model makes qualitative predictions regarding the conditions under which antibiotic resistance genes will be selected to be carried on plasmids, as opposed to on the chromosome. Specifically, we were interested in asking whether the intensity and interval between treatments will select for resistance to be on a plasmid, or on a chromosome. However, we find that our results generally hold under a wide range of different parameter ranges (see supplementary material for details). There is a wide range of literature available with which such models could be parameterised, and it would be possible to design models to test when selection will result in resistance to be carried on plasmids. For example, it is possible to add more realism to model the pharmacodynamics of drugs and how they kill bacteria [e.g. [40,48]], and there are some studies from which the costs of antibiotic resistance can be estimated [for example, see references in [49]]. Rates of horizontal gene transfer range widely and in the case of conjugation can range between 10^-3 ^and 10^-1 ^per donor in biofilms and more than 10^-5 ^in water or soil [50-52]. As plasmids have been described as the Achilles' heal of drug resistant bacteria [53,54], understanding the conditions under which antibiotic resistant genes are carried by plasmids could help to develop strategies to minimise the spread of resistance [53]. Developing and parameterising more explicit models in combination with laboratory studies, in contrast to the simple qualitative models described here, will be essential to developing treatments than minimise the rates of transfer.

The use of multiple antibiotics and antibiotic cycling has been proposed as a way to prevent the evolution of antibiotic resistance [e.g. [55]]. A previous study showed that plasmids could play a role in the acquisition of multiple drug resistance by repeated gene transfer [56]. Thus, plasmids may reduce the effect of antimicrobial cycling, as multiple resistance could be acquired more quickly. In the case of multidrug therapy, we would predict that the results of our model would hold, and longer intervals between treatment regimes would favour plasmid-carried resistance, which would in turn favour the evolution of multiple resistance.

Given the biological variability in the mechanisms of resistance, horizontal transfer and persistence, some of our assumptions may not hold in specific settings. Our work rests on costly antibiotic resistance and should thus apply particularly to mechanisms that need to be strongly expressed or that require a lot of cellular energy. When resistance proteins catalyse reactions that modify the antibiotic, such as in the case of β-lactamases, chloramphenicol acetyltransferase or aminoglycoside acetyltransferases, a higher expression level is expected to translate to a higher resistance level and the cell will have to pay high protein synthesis costs in order to be resistant. The same is true for efflux pumps, such as ABC -transporters or MF-type pumps [57]. The cost of resistance plasmids, including the cost of maintenance and expression, could be significantly ameliorated in experimental evolution experiments [58-60]. Based on the above arguments and the fact that even small costs are significant over evolutionary timescales, we expect there to be a large number of environments in which our findings would hold despite this effect, but it illustrates that with respect to the clinical view on this fundamental problem, more detailed case-by-case analyses will be required.

## Conclusions

To the best of our knowledge, our study represents the first model to explicitly examine the effect of treatment regimes plasmid-borne antibiotic resistance. We show that high transmission favours plasmid-carried resistance, in the absence of competing non-resistant plasmids. When other plasmids are present, low frequency treatments favour plasmid-carried resistance over chromosomal resistance, but a high intensity of antibiotic is needed for resistance plasmids to outcompete non-resistance plasmids. Targeting plasmid spread has been proposed to manage antibiotic resistance [54] and our results suggest that paying attention to the treatment regime is an essential requirement of any such strategy. The solution, as recommend by Paul Ehrlich almost a century ago, is to "hit hard and hit quickly" [61]. Gaining a quantitative understanding of the dynamics of plasmids will allow us to understand harmful patterns of antibiotic use more effectively.

## Authors' contributions

FS participated in the design of the study, performed analyses and wrote the paper. DJR conceived of the study, participated in the design of the study, performed analyses and wrote the paper. Both authors read and approved the final manuscript.

## Supplementary Material

Additional file 1 **The effect of antibiotic dosage intensity and the interval between treatments on the cell types persisting at equilibrium for the basic model in the absence of segregation (i.e. *s *= 0)**. "F" denotes wild-type cells, "P" denotes cells infected with a plasmid carrying resistance and "C" denotes cells with resistance on the chromosome. Plasmid transmission is *β *= 0.01 (in figure A) and *β *= 0.1 (in figure B). Cell types that are present in the population at a density greater than exceeding 0.001 are shown. The plots were calculated by running the simulation for a number of parameter values for 5,000 time-steps. Lines were then smoothed by interpolation. Parameters used are *r *= 1, *a *= 1, *c*_*c *_= 0.02, *c*_*p *_= 0.02, *x *= 0.05, *m *= 0.1, *s *= 0 and *l *= 0.5. Click here for file

Additional file 2 **The effect of antibiotic dosage intensity and the interval between treatments on the cell types persisting at equilibrium for the extended model, in the absence of segregation (i.e. *s *= 0)**. "F" denotes wild-type cells, "P" denotes cells infected with a plasmid carrying resistance and "C" denotes cells with resistance on the chromosome and "B" denotes cells carrying plasmids that do not code for resistance genes. Plasmid transmission is *β *= 0.01 (in figure A) and *β *= 0.1 (in figure B). Cell types that are present in the population at a density greater than exceeding 0.001 are shown. The plots were calculated by running the simulation for a number of parameter values for 5,000 time-steps. Lines were then smoothed by interpolation. Parameters used are *r *= 1, *a *= 1, c_c _= 0.02, c_p _= 0.02, *x *= 0.05, *m *= 0.1, *s *= 0 and *l *= 0.5. Click here for file

Additional file 3 **The effect of antibiotic dosage intensity and the interval between treatments on the cell types persisting at equilibrium for the extended model**. "F" denotes wild-type cells, "P" denotes cells infected with a plasmid carrying resistance and "C" denotes cells with resistance on the chromosome and "B" denotes cells carrying plasmids that do not code for resistance genes. Plasmid segregation *s *= 0.01 (in figure A) and *s *= 0.0001 (in figure B). Cell types that are present in the population at a density greater than exceeding 0.001 are shown. The plots were calculated by running the simulation for a number of parameter values for 5,000 time-steps. Lines were then smoothed by interpolation. Parameters used are *r *= 1, *β *= 0.1, *a *= 1, c_c _= 0.02, c_p _= 0.02, *x *= 0.05, *m *= 0.1 and *l *= 0.5. Click here for file

Additional file 4 **The effect of antibiotic dosage intensity and the interval between treatments on the cell types persisting at equilibrium for the extended model**. "F" denotes wild-type cells, "P" denotes cells infected with a plasmid carrying resistance and "C" denotes cells with resistance on the chromosome and "B" denotes cells carrying plasmids that do not code for resistance genes. The cost of antibiotic resistance is either *c*_*c *_= 0.04, *c*_*p*_= 0.02 (in figure A) or c_c _= 0.02, c_p _= 0.04 (in figure B), in the absence of segregation (i.e. *s *= 0). Cell types that are present in the population at a density greater than exceeding 0.001 are shown. The plots were calculated by running the simulation for a number of parameter values for 5,000 time-steps. Lines were then smoothed by interpolation. Parameters used are *r *= 1, *β *= 0.1, *a *= 1, *x *= 0.05, *m *= 0.1, *s *= 0 and *l *= 0.5. Click here for file

Additional file 5 **The effect of antibiotic dosage intensity and the interval between treatments on the cell types persisting at equilibrium for the extended model, in the absence of segregation (i.e. *s *= 0)**. In Figures A, C and E the degradation rate of the antibiotic is *l *= 0.1, while in Figures A, C and E, the degradation rate of the antibiotic is *l *= 5. In figures A and B the antibiotic induced mortality is *m *= 0.0001, in figures C and D the antibiotic induced mortality is *m *= 0.01 and in figures E and F the antibiotic induced mortality is *m *= 1. "F" denotes wild-type cells, "P" denotes cells infected with a plasmid carrying resistance and "C" denotes cells with resistance on the chromosome and "B" denotes cells carrying plasmids that do not code for resistance genes. Cell types that are present in the population at a density greater than exceeding 0.001 are shown. The plots were calculated by running the simulation for a number of parameter values for 5,000 time-steps. Lines were then smoothed by interpolation. Parameters used are *r *= 1, *a *= 1, *β *= 0.1, c_c _= 0.02, c_p _= 0.02, *x *= 0.05 and *s *= 0. Click here for file
