# Supplementary figures and images for: The evolution of plasmid-carried antibiotic resistance
Source: BMC Evol Biol. 2011 May 19;11:130. doi: 10.1186/1471-2148-11-130 (PMC3118148; doi:10.1186/1471-2148-11-130)

A

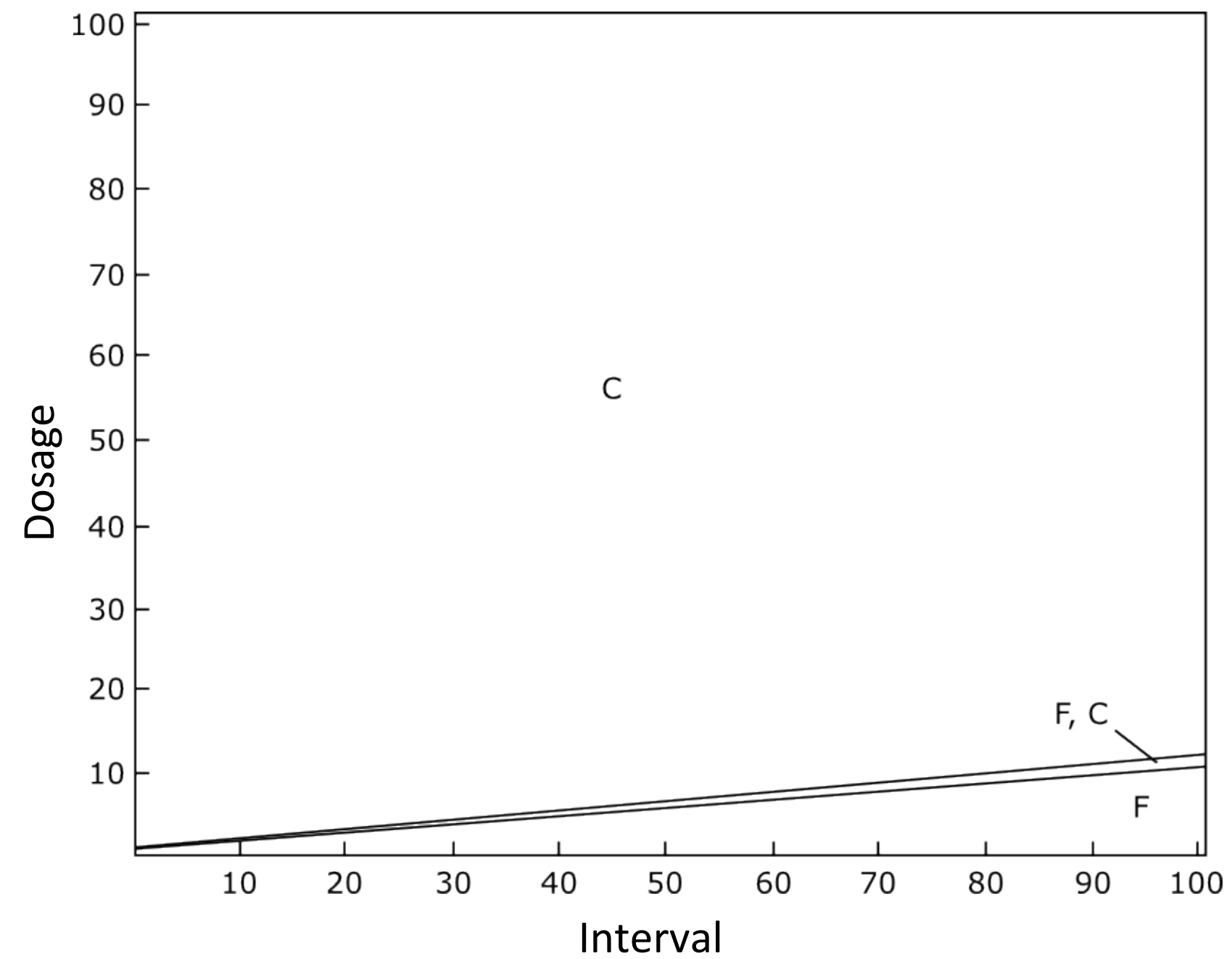

B

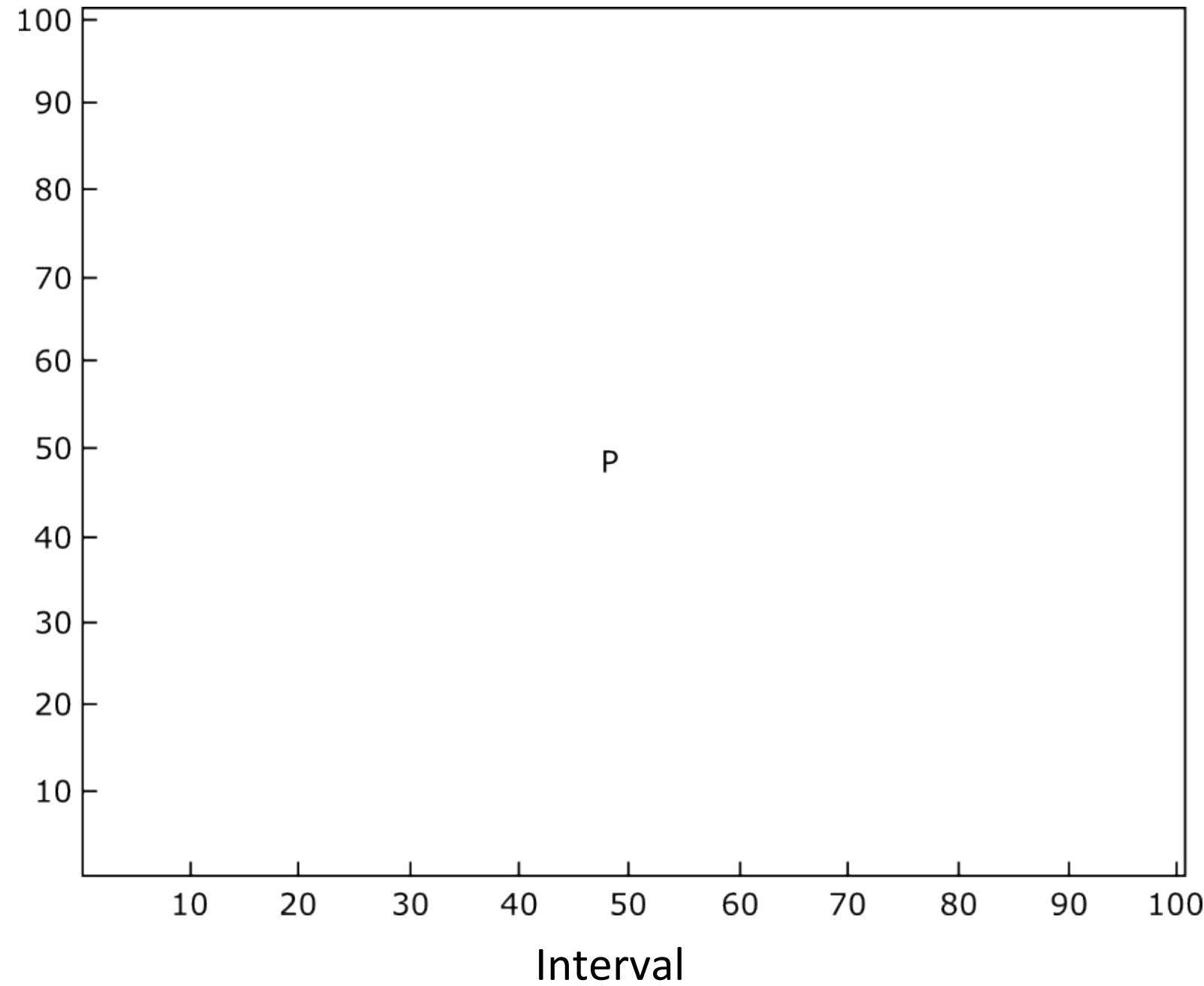

Supplement: Additional file 1 — The effect of antibiotic dosage intensity and the interval between treatments on the cell types persisting at equilibrium for the basic model in the absence of segregation (i.e. s = 0). "F" denotes wild-type cells, "P" denotes cells infected with a plasmid carrying resistance and "C" denotes cells with resistance on the chromosome. Plasmid transmission is β = 0.01 (in figure A) and β = 0.1 (in figure B). Cell types that are present in the population at a density greater than exceeding 0.001 are shown. The plots were calculated by running the simulation for a number of parameter values for 5,000 time-steps. Lines were then smoothed by interpolation. Parameters used are r = 1, a = 1, cc = 0.02, cp = 0.02, x = 0.05, m = 0.1, s = 0 and l = 0.5. [file 1471-2148-11-130-S1.PDF]

A

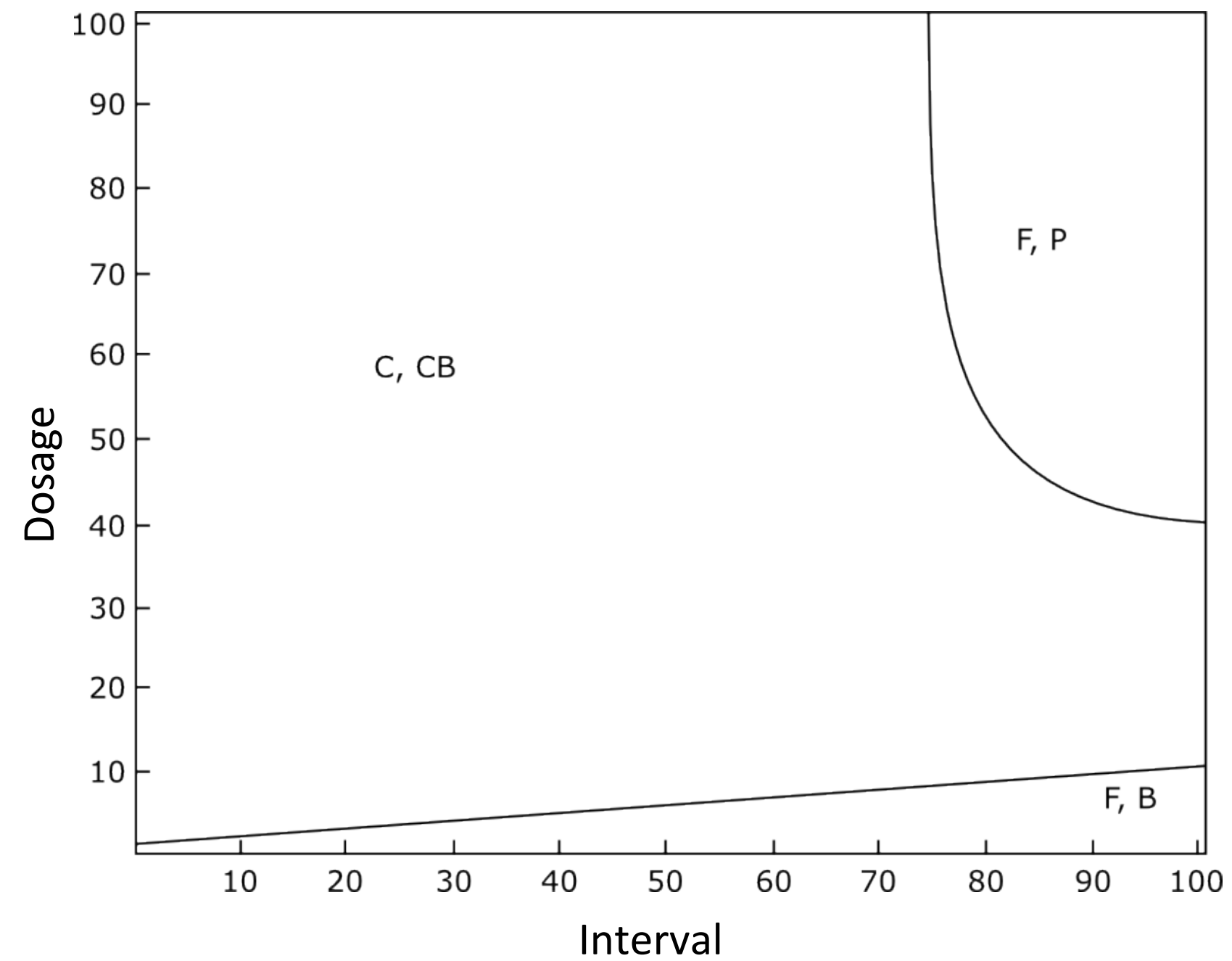

B

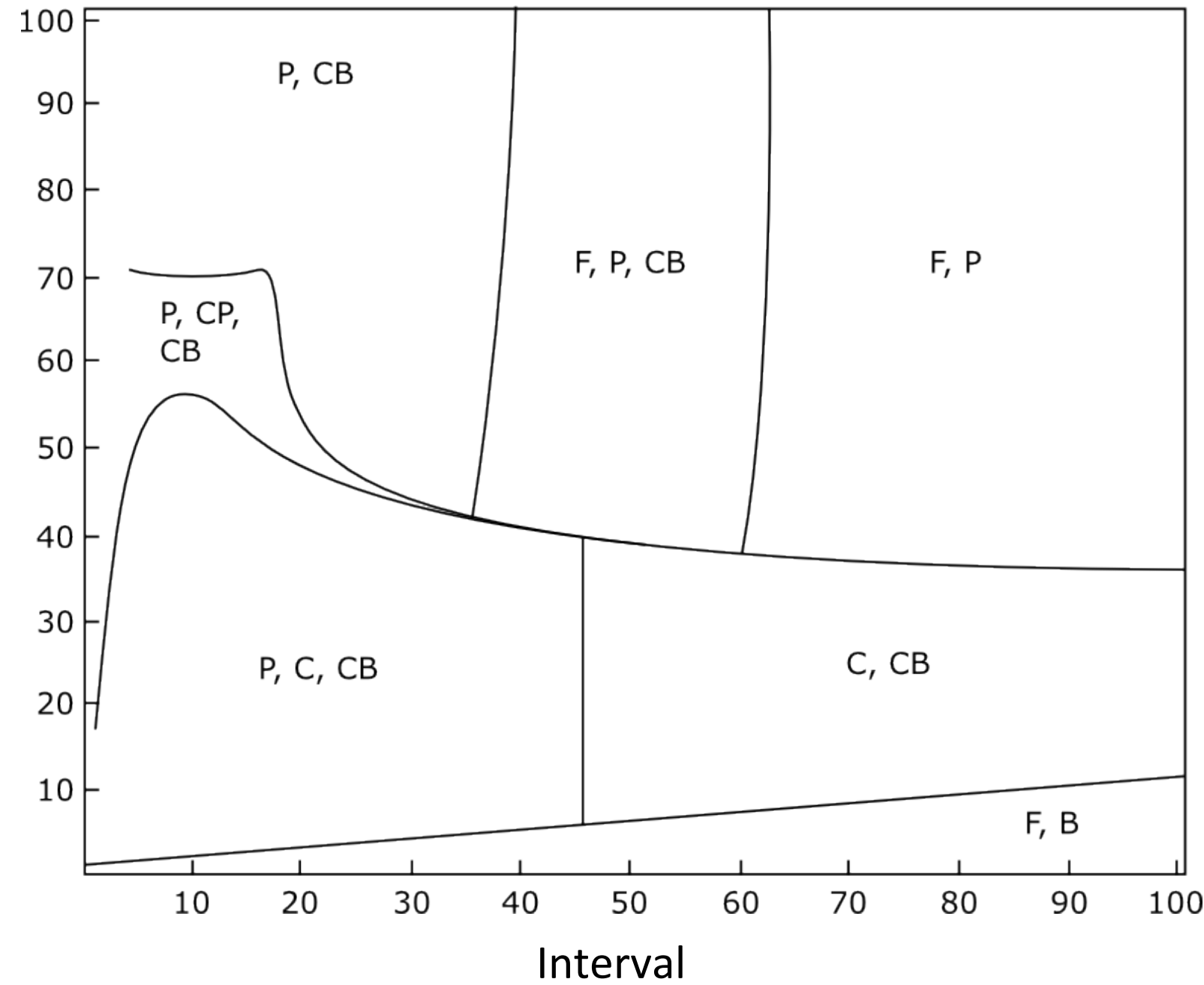

Supplement: Additional file 3 — The effect of antibiotic dosage intensity and the interval between treatments on the cell types persisting at equilibrium for the extended model. "F" denotes wild-type cells, "P" denotes cells infected with a plasmid carrying resistance and "C" denotes cells with resistance on the chromosome and "B" denotes cells carrying plasmids that do not code for resistance genes. Plasmid segregation s = 0.01 (in figure A) and s = 0.0001 (in figure B). Cell types that are present in the population at a density greater than exceeding 0.001 are shown. The plots were calculated by running the simulation for a number of parameter values for 5,000 time-steps. Lines were then smoothed by interpolation. Parameters used are r = 1, β = 0.1, a = 1, cc = 0.02, cp = 0.02, x = 0.05, m = 0.1 and l = 0.5. [file 1471-2148-11-130-S3.PDF]

**A**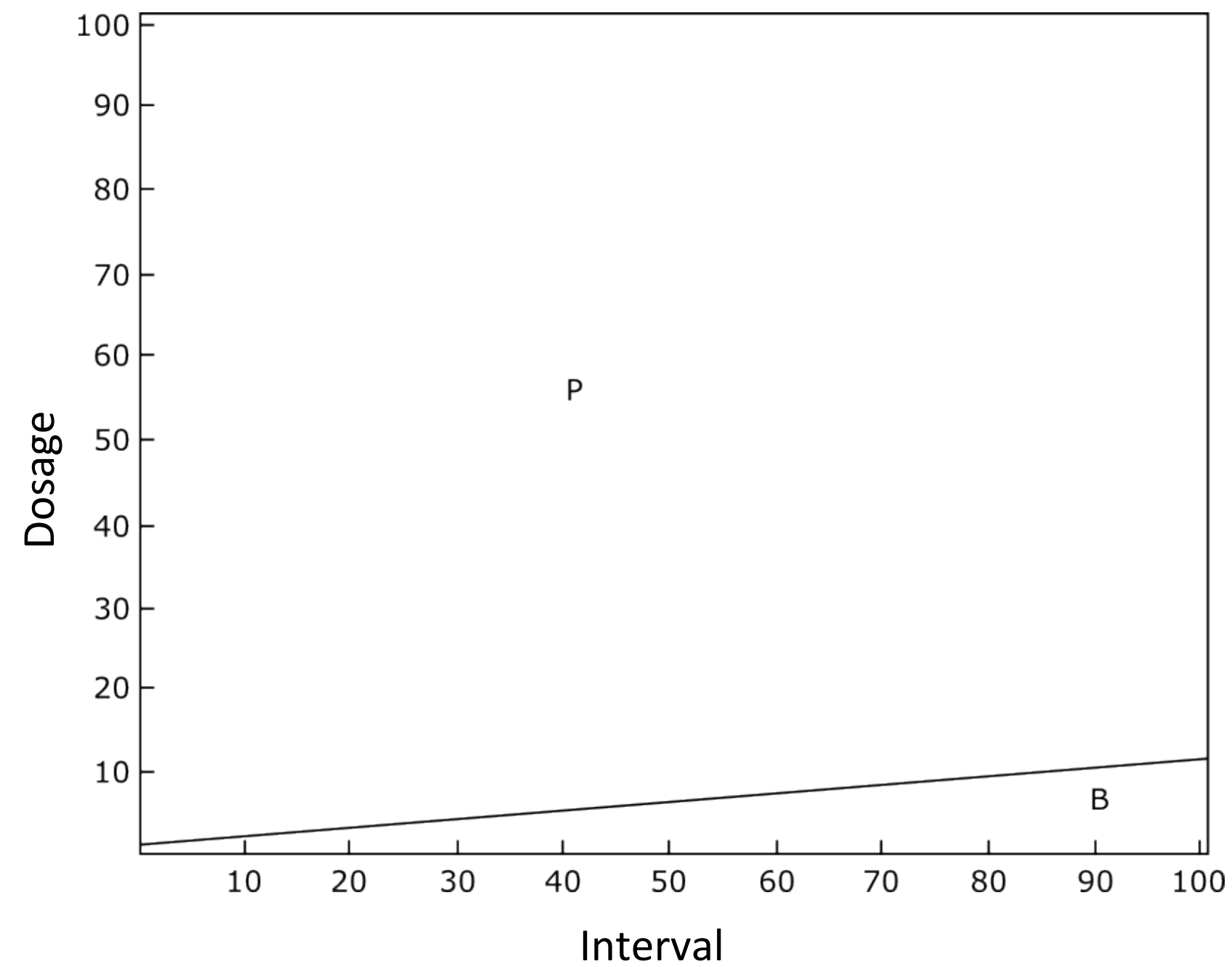**B**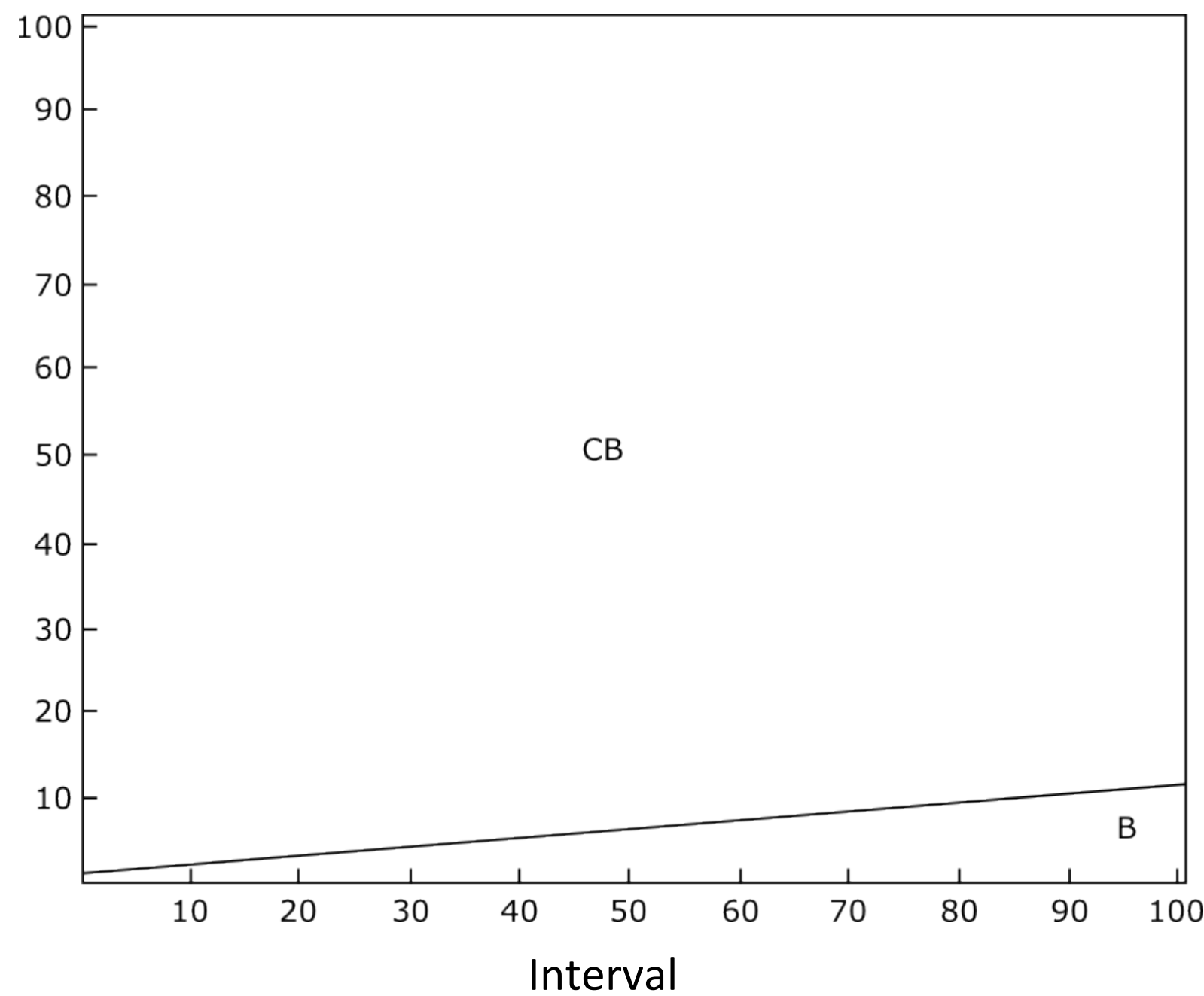

Supplement: Additional file 4 — The effect of antibiotic dosage intensity and the interval between treatments on the cell types persisting at equilibrium for the extended model. "F" denotes wild-type cells, "P" denotes cells infected with a plasmid carrying resistance and "C" denotes cells with resistance on the chromosome and "B" denotes cells carrying plasmids that do not code for resistance genes. The cost of antibiotic resistance is either cc = 0.04, cp= 0.02 (in figure A) or cc = 0.02, cp = 0.04 (in figure B), in the absence of segregation (i.e. s = 0). Cell types that are present in the population at a density greater than exceeding 0.001 are shown. The plots were calculated by running the simulation for a number of parameter values for 5,000 time-steps. Lines were then smoothed by interpolation. Parameters used are r = 1, β = 0.1, a = 1, x = 0.05, m = 0.1, s = 0 and l = 0.5. [file 1471-2148-11-130-S4.PDF]

**A**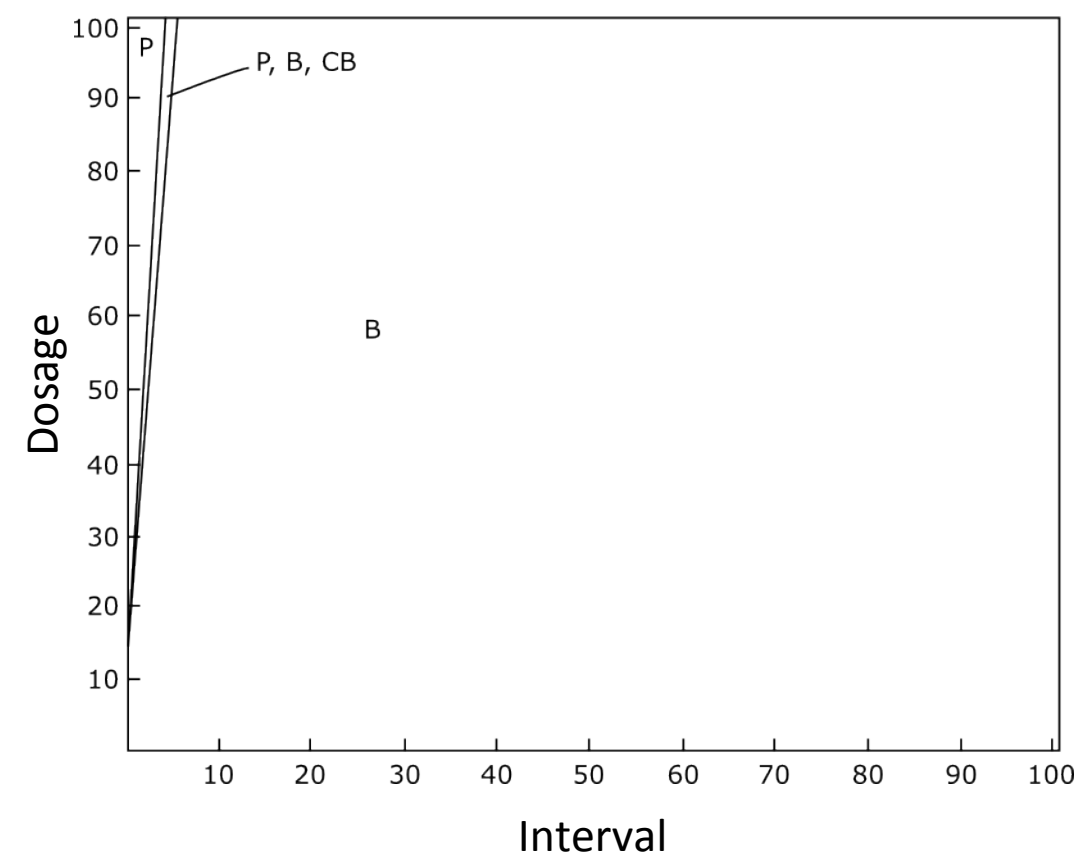**B**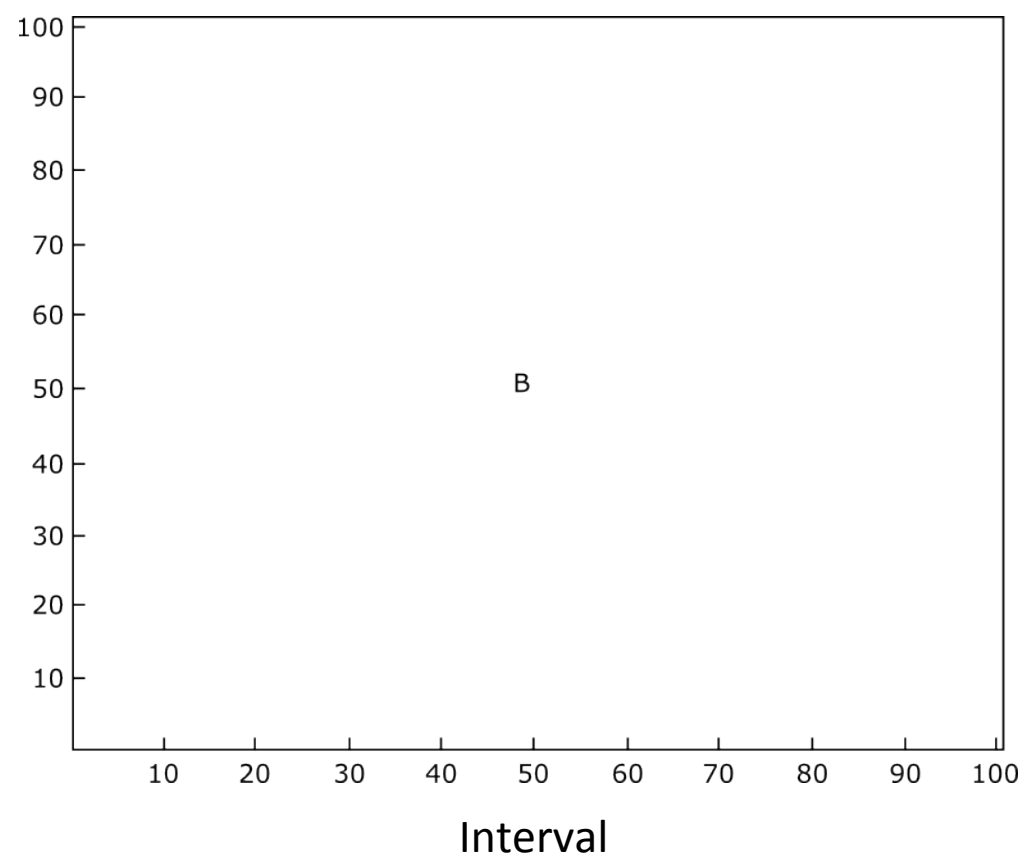**C**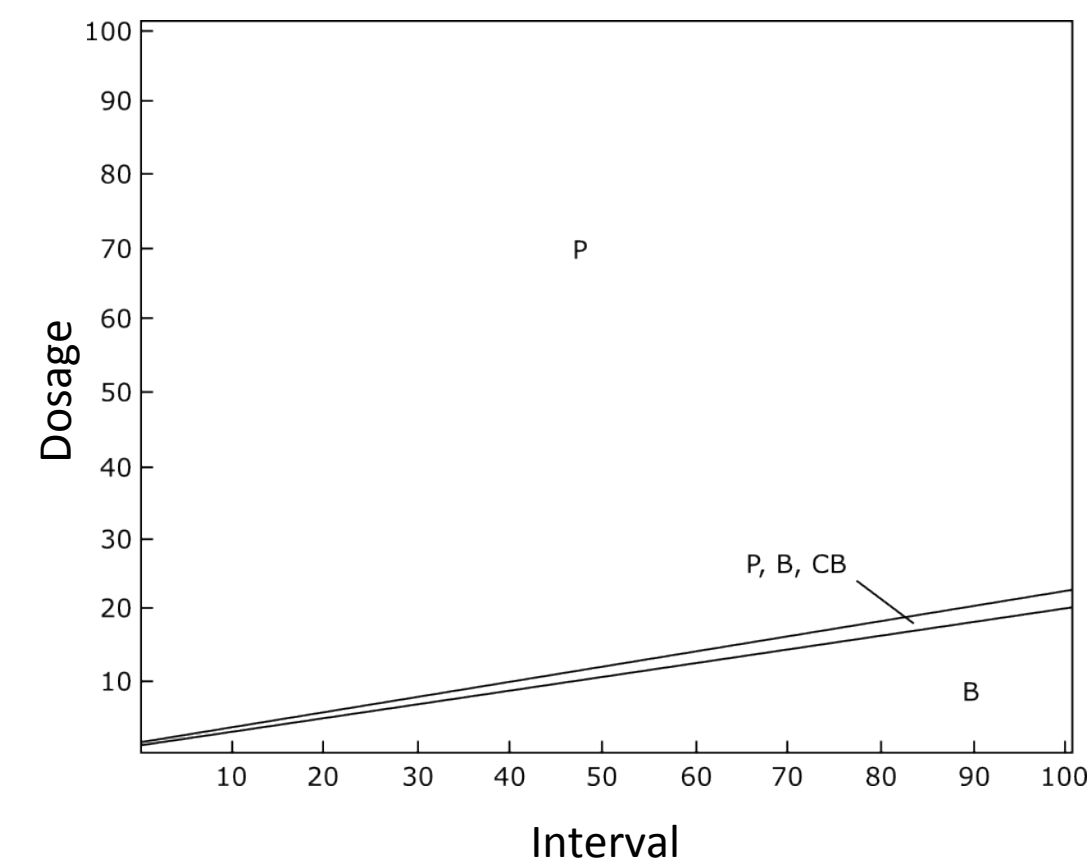**D**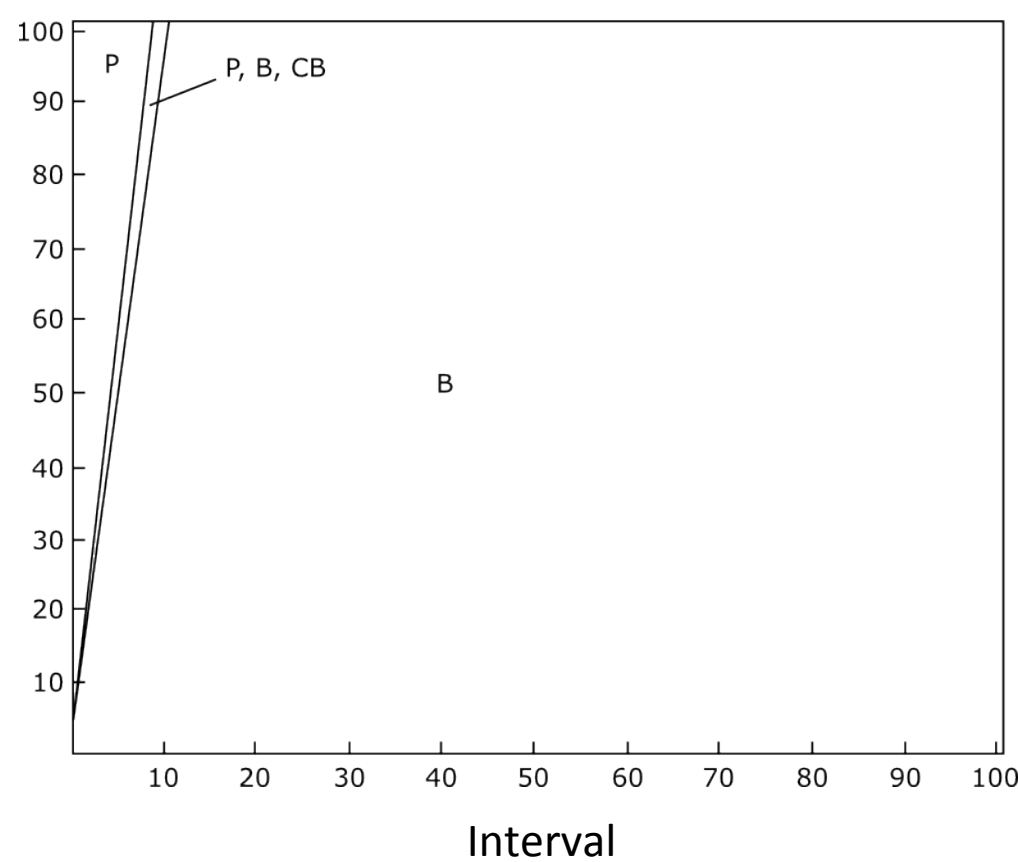**E**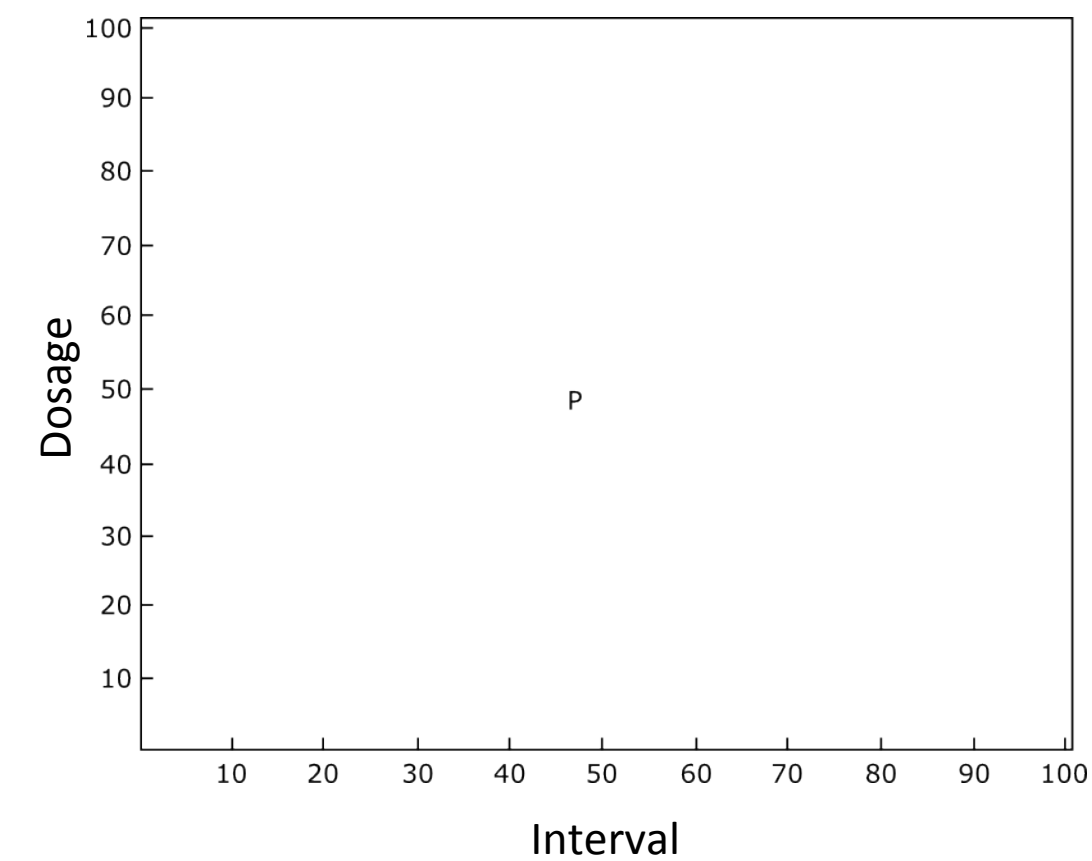**F**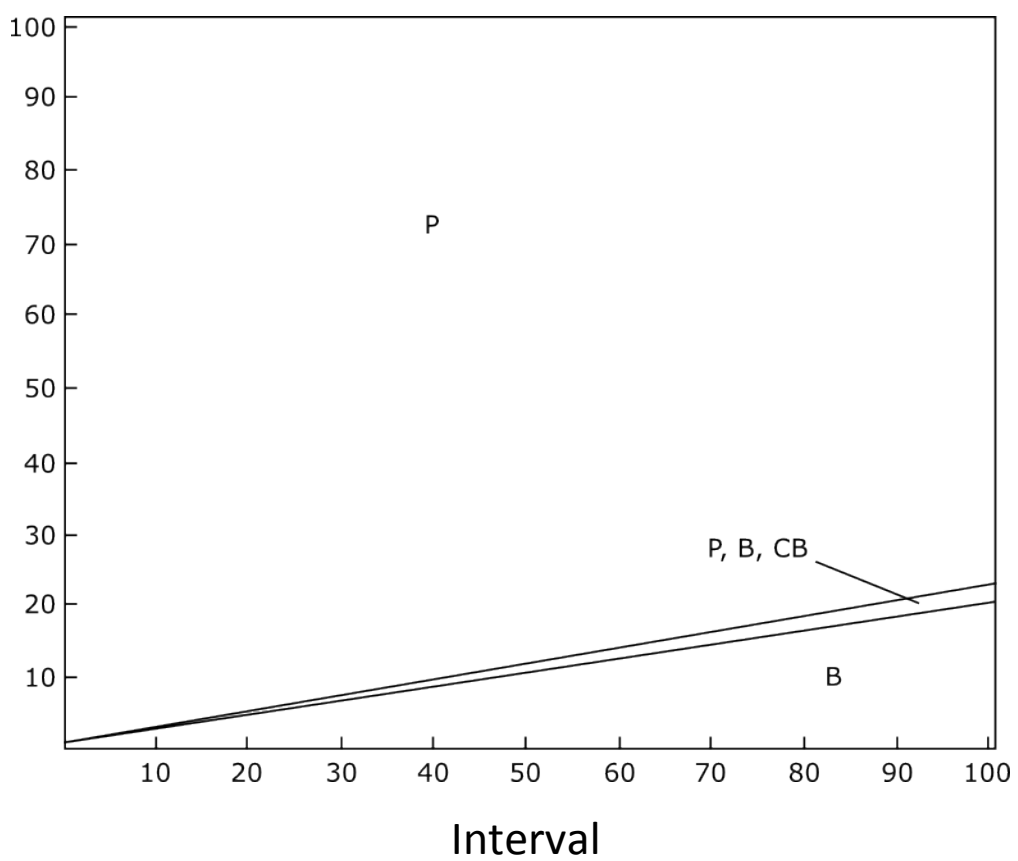

Supplement: Additional file 5 — The effect of antibiotic dosage intensity and the interval between treatments on the cell types persisting at equilibrium for the extended model, in the absence of segregation (i.e. s = 0). In Figures A, C and E the degradation rate of the antibiotic is l = 0.1, while in Figures A, C and E, the degradation rate of the antibiotic is l = 5. In figures A and B the antibiotic induced mortality is m = 0.0001, in figures C and D the antibiotic induced mortality is m = 0.01 and in figures E and F the antibiotic induced mortality is m = 1. "F" denotes wild-type cells, "P" denotes cells infected with a plasmid carrying resistance and "C" denotes cells with resistance on the chromosome and "B" denotes cells carrying plasmids that do not code for resistance genes. Cell types that are present in the population at a density greater than exceeding 0.001 are shown. The plots were calculated by running the simulation for a number of parameter values for 5,000 time-steps. Lines were then smoothed by interpolation. Parameters used are r = 1, a = 1, β = 0.1, cc = 0.02, cp = 0.02, x = 0.05 and s = 0. [file 1471-2148-11-130-S5.PDF]
